# The Potential of Plant Extracts Used in Cosmetic Product Applications—Antioxidants Delivery and Mechanism of Actions

**DOI:** 10.3390/antiox13111425

**Published:** 2024-11-20

**Authors:** Cristina-Ştefania Gǎlbǎu, Marius Irimie, Andrea Elena Neculau, Lorena Dima, Lea Pogačnik da Silva, Mihai Vârciu, Mihaela Badea

**Affiliations:** 1 Faculty of Medicine, Transilvania University of Brasov, Romania, No. 56, Nicolae Bǎlcescu St., 500019 Braşov, Romania; cristina.adochite@unitbv.ro (C.-Ş.G.); marius.irimie@unitbv.ro (M.I.); andrea.neculau@unitbv.ro (A.E.N.); lorena.dima@unitbv.ro (L.D.); mihai.varciu@unitbv.ro (M.V.); 2 Biotechnical Faculty, University of Ljubljana, Jamnikarjeva 101, 1000 Ljubljana, Slovenia; lea.pogacnik@bf.uni-lj.si

**Keywords:** plant extracts, cosmetics, antioxidants, bioactive compounds

## Abstract

Natural ingredients have been used in skincare products for thousands of years. The current focus is on novel natural bioactivities that shield the skin from UV rays and free radicals, among other damaging elements, while enhancing skin health. Free radicals significantly contribute to skin damage and hasten ageing by interfering with defence and restorative processes. Plants contain natural chemicals that can scavenge free radicals and have antioxidant capabilities. Plant materials are becoming increasingly popular as natural antioxidants related to the expanding interest in plant chemistry. This review focuses on the significance of medicinal plants in skin health and ageing and their potential as a source of antioxidant substances such as vitamins, polyphenols, stilbenes, flavonoids, and methylxanthines.

## 1. Introduction

For many years, skincare products have been made with natural components, whether they come from mineral, animal, or plant origins [1,2,3]. In this century, the use of naturally occurring chemicals continues to increase, probably due to the social media influence. Between 2015 and 2019, the global market for naturally made cosmetics was growing by 10–11% per year. This market also offers a substantial opportunity for the cosmetics industry because many consumers are willing to pay more for these products [4,5]. Plant-based products may be used topically to treat a variety of skin conditions and for skin care. In addition to being more ecologically friendly than traditional cosmetics, cosmetics enhanced with bioactive ingredients are well adapted to the demands of the skin. Plant extracts, which are a rich source of physiologically active chemicals that have a substantial impact on human skin, are a class of natural compounds that are often utilized in cosmetics. We have to emphasize that there are a limited number of studies concerning substances added to food that had an effect on the skin. In some cases, scientific evidence is lacking and the effect is only an assumption.

Many factors, including environmental exposure, gut microbes, stability, activity, and variability in endogenous chemical levels that modulate biotransformation pathways, can influence an organism’s metabolism. Antioxidant phytochemicals including vitamins, such as vitamin E, vitamin A, and vitamin C, and polyphenols, tocopherols, and carotenoids have been shown to enhance our aesthetic well-being. These phytochemicals have anti-inflammatory, antioxidant, photoprotective, anti-ageing, antiviral, and antibacterial characteristics. Synergistic stabilizing effects have been demonstrated when combining synthetic and natural antioxidants [6]. Antioxidants include both enzymatic and non-enzymatic compounds [7]. Their distribution is frequently influenced by the various skin cell types. For example, melanocytes lack antioxidant enzymes [8]. Antioxidants are classified as biopharmaceuticals according to their permeability and solubility [9].

According to estimates, there are three different types of antioxidants: low solubility—low permeability, low solubility—high permeability, and high solubility—low permeability (vitamin C is present in cellular fluids and vitamin E is found in cell membranes). When used in topical treatments, water solubility, restricted permeability, and instability are the primary concerns. The instability caused by external stresses (such as air, light, moisture, heat, oxygen, etc.) affects the product’s shelf life [6]. Due to their restricted permeability and water solubility [10], they have limited possibilities of entering deeper epidermal layers and reaching the target location. Antioxidant delivery systems must be capable of being absorbed into the food or beverage matrix without affecting the end product’s appearance, texture, flavour, or shelf life [11]. Throughout manufacturing, storage, transit, and usage, it must tolerate environmental stresses like thermal processing, exposure to light, dynamic agitation, cooling, freezing, and dehydration [11]. This study aims to underline the physicochemical importance of antioxidant compounds used in cosmetics, considering their delivery and mechanism of action, summarizing the novelty of the results studied in vitro and in vivo.

## 2. Materials and Methods

### 2.1. Search Strategy

Since most connected papers and themes were published recently, our research included all studies published in PubMed, Scopus, and a manual Google Scholar search. The keywords “antioxidants” AND (“dermatology” OR “inflammation” OR “cosmetics” OR “proliferation”) formed the basis of the scientific literature search approach. Significant publications were chosen based on different plants’ biological, chemical, and functional characteristics.

### 2.2. Inclusion and Exclusion Criteria

Following criteria, like experimental and review studies, full articles for each selected abstract were retrieved for review. All English-language research articles were included. Investigations were based on in vivo and in vitro research publications. Research publications dealing with specific plant extracts were included. Still, those that considered a combination of plant extracts or a formulation of some other chemical ingredients were excluded. The CAS numbers of the compounds were mentioned in the article, providing valuable information and facilitating the future search for the classification and labelling in the database.

## 3. Results

### 3.1. Vitamins

Plants produce compounds (phytochemicals) through their secondary metabolism that can protect them from pests, bacteria, and atmospheric pollutants. In both people and animals, some of these compounds (such as polyphenols, cysteine sulphoxides, and carotenoids) can be combined with free radicals to create stable chemical species [12]. Numerous biological effects of phytochemicals which are beneficial to human health include photoprotective, anti-ageing, anti-inflammatory, antibacterial, antiviral, and anticancer activities [12]. Vitamins E, C, and A, for example, have the potential to be antioxidants and have skincare benefits (Table 1). Collagen synthesis is controlled by vitamin C. Free radicals are actively neutralized by vitamin E, which also helps to soften the skin [13]. Stretch marks, burn scars, and new skin cell growth are reduced by vitamin A, which also boosts collagen formation [14,15].

#### 3.1.1. Vitamin A

Beta-carotene (pro-vitamin A), vitamin A and its derivatives, and other ingredients have been used as cosmetic additives. Beta-carotene can be found in foods like tomatoes, carrots, and yellow vegetables, whereas the main animal sources of vitamin A are liver and egg yolk. Beta-carotene and vitamin A were also found to be photoprotective by decreasing the quantity of peroxyl lipid radicals in the skin of mice exposed to UV radiation [16]. However, because beta-carotene is so fragile, other types of vitamin A are frequently included in cosmetic compositions. The capacity of vitamin A (CAS number: 68-26-8) and its derivatives to correct keratinization is the main advantage of these ingredients in the cosmetics industry. Tretinoin (CAS number: 302-79-4), vitamin A alcohol (retinol), vitamin A esters (retinyl palmitate (CAS number: 79-81-2), retinyl acetate (CAS number: 127-47-9)), and vitamin A aldehyde (retinal) (CAS number: 116-31-4) are some of the common vitamin A compounds that can be found in cosmetics. These are present in cosmetic compositions in various concentrations due to their involvement in controlling epithelial cell proliferation and differentiation [17].

#### 3.1.2. Vitamin C

Vitamin C (CAS number: 50-81-7), or ascorbate, is a hydrosoluble vitamin found in vegetables and citrus fruits. Its antioxidant properties and role as a cofactor in collagen hydroxylation events make it an essential nutrient. Since humans cannot produce ascorbate, nutritional intake is crucial. The capacity of vitamin C to immediately quench UV-induced free radicals and replenish vitamin E, another effective antioxidant, contributes to its popularity as a cosmetic element [18]. To maximize UV protection, combining sunscreen with a topical antioxidant is essential. Vitamin C does not absorb UV radiations but protects them by radical scavenging, in contrast to sunscreens, which do not [19]. Under laboratory circumstances, 10% topical vitamin C treatment reduced UVB-induced erythema by 52% and sunburn cell development by 40 to 60% [20]. Due to its capacity to promote collagen formation, vitamin C is also used as a component of anti-ageing products. Ascorbyl palmitate (CAS number: 137-66-6) [21], magnesium ascorbyl phosphate (CAS number: 114040-31-2) [22], and L-ascorbic acid (CAS number: 50-81-7) [23] are the three primary forms of ascorbic acid that are frequently found in cosmetics.

#### 3.1.3. Vitamin E

Vitamin E (CAS number: 59-02-9) is a liposoluble vitamin found in various foods, especially soybeans, nuts, wholemeal flour, and oils [24]. It is claimed that systemically reducing lipid peroxidation has several health advantages for the eyes and cardiovascular system. Numerous dermatological benefits of topically administered substances have been demonstrated. The powerful antioxidant properties of vitamin E serve as the main mechanism of action to support its significance. The “protective” term has been employed to characterise the protective effects of vitamin E and its derivatives due to its ability to scavenge free radicals, specifically lipid peroxyl radicals. Numerous studies have demonstrated their capacity to lessen erythema and edema, sunburn cell development, and lipid peroxidation caused by UV radiation [25]. Reduced skin wrinkling and skin tumour growth have been linked to clinical improvement in the obvious indications of skin ageing [26].

#### 3.1.4. Coenzyme Q10

Coenzyme Q10 (CAS number: 303-98-0) is a botanical food ingredient, and its derivatives are used in functional foods and nutritional supplements. The antioxidant properties of coenzyme Q10 have been correlated with the speed-up in recovery of ATP levels following radiation in human fibroblasts and maintaining the stability of cellular energy levels in human keratinocytes. It prevents the harmful effects of photoaging, minimizes wrinkles, and improves skin smoothness on human skin [27]. It is an internal lipophilic molecule that is essential or useful for mitochondrial strength biotransformation and effective for antioxidants and human health [28]. Another study [29] described the effects of administrations of biological and adjuvant coenzyme Q10 therapy, which showed an association between the Psoriasis Area Severity Index (PASI) and the Dermatology Life Quality Index (DLQI) (*p* = 0.000132), which means that the daily administration of 100 mg coenzyme Q10 supplements to psoriatic patients for 12 weeks improved the correlation between PASI and DLQI. Coenzyme Q10 inhibited the deterioration of skin viscoelasticity, decreased the depth of microrelief lines (wrinkles), and enhanced the skin’s smoothness and fairness [30].

### 3.2. Polyphenols

Polyphenols are structured by one or more aromatic rings containing one or several hydroxyl groups. Depending on the number of phenolic rings and the elements that make up the structures linking these rings, it is possible to distinguish between various classes, such as phenolic acids, flavonoids, stilbenes and lignans [31].

Oral consumption of polyphenols has been related to several health benefits. However, their bioavailability can be limited and is mostly influenced by their chemical structure. The bioavailability is mainly determined by the amount of nutrients ingested, absorbed, and used in metabolic processes [32,33]. The various biological activities of polyphenols reflect the diversity of their structure [34]. They are recognized for their antioxidant, anti-inflammatory, antibacterial, antifungal, antiviral, anti-allergenic, anticancer, and anti-coagulant effects. Plant polyphenols are considered significant for maintaining healthy skin because of their effects on hydration, smoothness, softness, calming, and astringency [35,36,37]. Collagenase, elastase, and hyaluronidase, which catalyse the degradation of collagen and elastin fibres and hyaluronic acid, respectively, are all skin-specific enzymes that are inhibited by polyphenols. Additionally, they calm inflammation and lessen skin redness while promoting quicker epidermal regeneration, stabilizing capillaries, enhancing microcirculation, increasing skin suppleness, and shielding against damaging environmental factors like UV radiation. Antioxidants have been demonstrated to be associated with a decreased incidence of ROS-induced photoaging [38]. According to Khlebnikov et al. [39], antioxidants are “any substance that directly scavenges reactive oxygen species (ROS) or indirectly acts to upregulate antioxidant defences or inhibit ROS production”. The removal of radicals through direct interactions, scavenging, or the reduction of free radicals (such as hydroxyl, superoxide, peroxide, and alcoxyl radicals) to less reactive molecules is the basis for polyphenols’ antioxidant and antiradical effects. Additionally, polyphenols can chelate heavy metal cations (such as Cu^2+^ and Fe^2+^), blocking the Fenton reactions (which result in the production of the highly reactive hydroxyl radical ^•^OH) and limiting the activity of numerous free radical-producing enzymes (xanthine oxidase, protein kinase, and lipoxygenase). Other antioxidants, such as ascorbate in the cytosol or tocopherol in biological membranes, are also stimulated and protected due to their activity [40]. Pure polyphenolic substances interact well with other antioxidants to delay skin ageing. An oral antioxidant combination of pycnogenol, evening primrose oil, vitamin C, and vitamin E was studied by Cho at al. [41] for its impact on UVB-induced wrinkle formation. According to the investigation, administering antioxidants to hairless mice exposed to UVB radiation three times per week for 10 weeks dramatically reduced the UVB-induced production of matrix metalloproteinases, mitogen-activated protein kinase, and transcription factor AP-1. In addition, TGF-2 and type I procollagen expression was increased. According to scientific studies, oral treatment with the antioxidant mixture can reduce the appearance of wrinkles by reducing matrix metalloproteinase expression and boosting collagen synthesis [40]. Pomegranates (*Punica granatum*) are a very good source of polyphenols (anthocyanins and hydrolysed tannins) that have beneficial effects on skin conditions [38]. Pomegranate extract has been shown to have photochemoprotective, antioxidant, anti-inflammatory, and anti-proliferative effects. Pomegranate fruit extract has been proven to promote skin colour and restore brightness to skin exposed to UV radiation [42] and minimize UVB-induced oxidative stress and the oxidation of skin proteins [43].

### 3.3. Stilbenes

The most important stilbenes found in grapes are *cis*- and *trans*-resveratrol (3,5,4′-trihydroxystilbene) (CAS number: 501-36-0), resveratrol-3-O-β-D-glucopyranoside (piceid) (CAS number: 27208-80-6), piceatannol (3,4,3′,5′-tetrahydroxy-trans-stilbene) (CAS number: 10083-24-6) [44] and viniferins which are resveratrol dimers [45]. Research on the anti-carcinogenic, antioxidant, and anti-melanogenesis properties of natural stilbenes against ultraviolet light radiation were performed [46] (Table 2).

#### 3.3.1. Resveratrol

Resveratrol (CAS number: 501-36-0) is the main stilbene found naturally in grapes [47]; it is noted for its anticancer, antioxidant, anti-inflammatory and cardioprotective properties [48]. According to De Filippis et al. [49], resveratrol has a strong antioxidant activity on molecular targets related to tumour initiation, promotion, and progression [50]. In turn, it is proposed that it can initiate apoptosis (by regulating and modulating the p53 protein responsible for tumour destruction, by depleting levels of Bcl-2 and Bcl-xL anti-apoptotic molecules and by interfering with the process of nuclear transcription moderated by NF-κB and AP-1 cascades) [51] and reduction of angiogenesis through inhibition of FGF-2 and VEGF, neovascularisation, as well as modulation of several signalling pathways linked to malignant progression or cell survival [52]. 

Its demonstrated capacity to permeate the skin barrier and anti-ageing properties are the main reasons for its prominence in dermatology and cosmetology. Resveratrol-containing formulations have been shown to promote fibroblast proliferation and raise the content of collagen III. Because of its affinity for the ERα and ERβ estrogen protein receptors, resveratrol helps to stimulate the formation of collagen types I and II. Furthermore, resveratrol also has antioxidant qualities, which means that by lowering the expression of AP-1 and NF-kB proteins and delaying the process of skin photoaging, it may shield cells from oxidative damage brought on by free radicals and UV radiation [53].

#### 3.3.2. Piceatannol

Astringenin, as piceatannol is also known, which is part of *trans*-resveratrol (trans-3,4,3′,5′-tetrahydroxystilbene), can be found naturally in red wine, sugar cane, grapes, berries, peanuts, and white tea [54]. Both resveratrol and piceatannol can induce direct antioxidant effects by scavenging free radicals and protecting proteins from cysteine groups under the effect of oxidative stress. A study described that the miR-181a was significantly downregulated in melanoma cancer tissues compared to their neighbouring ones, and strongly overexpressed in both WM266-4 and A2058 cells treated with piceatannol. Therefore, we propose that the apoptotic impact of piceatannol in melanoma cells may be associated with a high level of miR-181a expression [55].

#### 3.3.3. Pinosylvin

The natural polyphenol known as pinosylvin (3,5-dihydroxy-trans-stilbene) (CAS number: 22139-77-1) is a *trans*-stilbenoid and is found in pine trees, specifically in *Pinus sylvestris* [56]. A study in male mice confirmed the action of pinosylvin in reversing the agonist effect of the transient receptor potential ankyrin 1. High concentrations of pinosylvin (100 μM) showed less effect on the activation of the transient receptor potential ankyrin 1 (TRPA1), thus confirming its anti-inflammatory potency. Also, in the same mice, doses of pinosylvin decreased interleukin-6 levels [57].

#### 3.3.4. Pterostilbene

A natural analogue of resveratrol is pterostilbene (3,5-dimethoxy-4′-hydroxystilbene) (CAS number: 537-42-8), which has greater antioxidant activity than resveratrol and, therefore, has great potential for use in the clinical treatment of various diseases [58]. According to scientific studies [59], it has shown strong chemopreventive properties and beneficial effects of pterostilbene, similar to resveratrol in several in vitro and in vivo studies with different types of cancer. 

Pterostilbene is an active apoptotic constituent and can inhibit growth, adhesion, and metastatic growth [60]. These qualities have been reported in various cancer research [61], including breast cancer, pancreatic cancer, stomach cancer, and colon carcinoma [62].

### 3.4. Phenolic Acids

More than one-third of dietary phenols are phenolic acids. They are naturally occurring in plants as free polyphenols or bound; the latter are linked by ester, ether or acetal bonds [63]. Phenolic acids are made up of a diverse group of chemical substances, where more than 8000 different components can be found that influence human and animal diets. One of their main properties is that they can donate hydrogen molecules or chelate iron and copper ions, preventing low-density lipoproteins from oxidising [64]. They are closely linked to reducing the risk of neurodegenerative diseases, cardiovascular diseases, gastrointestinal [65], colon, breast or ovarian cancer, leukaemia [66], increasing bile secretion, decreasing cholesterol levels, decreasing blood lipid levels, and antimicrobial activities [67].

Phenolic acids can be found in edible vegetables, fruits and nuts suitable for the human diet, with strong anti-diabetic properties, consumption of which reduces the risk of diabetes by regulating the key pathway of carbohydrate metabolism and hepatic glucose homeostasis, including glycolysis, glycogenesis, and gluconeogenesis [68]. Structurally, phenolic acids are derived from the hydroxylation of cinnamic acid [69] or benzoic acid. The phenolic acids most recognized in human foods are caffeic and ferulic acids [70]. Although they are considered direct antioxidants, they also exhibit indirect antioxidant properties by producing endogenous protective enzymes and positive regulatory effects on signalling pathways [71].

According to Drawbridge et al. [72], cereals possess among their phytochemical components phenolic acids that have antioxidant and anti-inflammatory effects. The phenolic acids commonly found in cereals are p-hydroxybenzoic (CAS number: 99-96-7), protocatechuic (CAS number: 99-50-3), vanillic (CAS number: 121-34-6), gallic (CAS number: 149-91-7), syringic (CAS number: 530-57-4), caffeic (CAS number: 331-39-5), p-coumaric (CAS number: 231-000-0), ferulic (CAS number: 1135-24-6), and sinapic acids (CAS number: 530-59-6) (Table 3). Lodovici et al. [73] suggest that daily intakes of hydroxybenzoic and hydroxycinnamic acid range from 11 mg/day to 211 mg/day. In contrast, caffeic acid intake is about 206 mg/day in subjects who consume coffee. In another study, the presence of the gentisic and ferulic acids were reported in the roots of *Brassica rapa* ssp. Pekinensis [74]. The concentrations of these compounds were 0.68 mg/g and 0.56 mg/g after elicitation with copper nanoparticles.

Okafor et al. [75] reported a range of hydroxybenzoic acids in different Bambara groundnut (*Vigna subterranean*) varieties, where 4-hydroxybenzoic acid (p-hydroxybenzoic acid), 2,6-dimethoxybenzoic acid, protocatechuic acid, caffeic acid, and ferulic acid were found in the highest quantity [76].

### 3.5. Flavonoids

The chemical composition of flavonoids is 2-phenyl-benzo-a-pyrones. In their natural mode, it is possible to find various patterns in the composition of the two benzene rings that form the basic structure of this compound [77]. Depending on the connection between the rings and the ring structures, in addition to the various hydroxylation and glycosylation patterns, flavonoids can be classified into different subclasses as the following: flavones, flavonols, flavanols, flavanones, isoflavones, and anthocyanins (Table 4) [78,79].

#### 3.5.1. Flavones

This is a subclass that features a double bond between the C2 and C3 of the rings, and a ketone at C4 [80], but they are capable of containing other substituents depending on the taxonomical characteristics of the plant. They can be hydroxylated, methylated, glucosylated, or alkylated [81]. Flavones can be both of natural and synthetical origin. In their natural form, they can be found in various foods and plant tissues, such as flowers, fruits, grapes, apples, celery, mint, and tea, among others [82].

An article written by Maher [83] describes the ability to increase performance and working memory in 12–15-month-old mice by intraperitoneal injections of 7,8-dihydroxyflavone flavone (5 mg/kg) over 10 days.

#### 3.5.2. Flavonols

This subclass of polyphenols is the most diverse in the plant kingdom and possesses strong physiological activity. Flavonols are secondary metabolites present in a wide variety of fruits, vegetables, and plants [84]. 

According to Nagula and Wairkar [85], human skin is commonly subjected to oxidative stress due to the influence of UV radiations, ozone radiation and other harmful substances. The main characteristics of flavonols include their ability to act as oxidising agents and protection against the formation of reactive oxygen species [85]. 

On the other hand, Farhadi et al. [86] state that the flavonols with high antimicrobial activity include quercetin (CAS number: 117-39-5), myricetrin (CAS number: 529-44-2), morin (CAS number: 654055-01-3), galangin (CAS number: 548-83-4), entadanin, rutin (CAS number: 153-18-4), and piliostigmol. The authors reported strong antimicrobial activity against *Porphyromonas gingivalis* in an in vitro investigation of some of these flavonols, with quercetin at a concentration of 0.0125 μg/mL showing the best results.

#### 3.5.3. Flavanols

Flavanols are found in significant amounts in various fruits and fruit products, such as juices, red wine, cocoa, and tea, among others. The absorption of flavanols in the human diet is limited, because parts of the fruits such as the hulls or seeds are discarded during processing or ingestion [87].

Gómez-Juaristi et al. [88] investigated the absorption and flavanol metabolism in two different soluble cocoa products, one with high flavanol content and one traditional, where for both a 35% absorption capacity was obtained, demonstrating that they are moderately bioavailable and considerably metabolised by the colonic microbiota.

In contrast, another article written by Geng et al. [89] reported on the antidepressant capacity of the flavanols catechin and epicatechin, originating from *Uncaria rhynchophylla*, which influenced melatonin receptors, by evaluating catechin metabolic pathways in mouse plasma.

#### 3.5.4. Flavanones

Flavanones are formed by a chain saturated by three carbon atoms and one oxygen atom and are constituted especially by naringin and hesperidin glycosides, which are the main compounds of citrus fruits and citrus peels, with a strong antioxidant and free radical inhibition capacity [90]; they are also found in tomatoes and a few aromatic plants such as mint [91].

Anacleto [92] evaluated the protective capacity of flavanones (naringenin) in pancreatic β-cells under oxidative stress, due to its anti-inflammatory and antioxidant capacities.

#### 3.5.5. Isoflavones

Isoflavones are found entirely in legumes and although they are not steroids, they have structural similarities to estrogens and pseudohormonal properties, which is why they are considered phytoestrogens [93]. Isoflavones can be hydrolysed through the gastrointestinal tract but mainly in the jejunum mediated by the collaboration of the brush border membrane and bacterial β-glucosidases [94], releasing aglycones which are absorbed into the intestinal epithelium [95].

Yonekura-Sakakibara et al. [96] proposed that the initial step in the biosynthesis of isoflavones is through the catalysis of 2-hydroxyisoflavanone synthase; isoflavone synthase transforms liquiritigenin and naringenin into 2-hydroxyisoflavanones, and then through dehydration of these are transformed into isoflavones by the influence of 2-hydroxyisoflavanone dehydratase.

#### 3.5.6. Anthocyanins

The pigments from which plants, flowers and fruits obtain their colours are anthocyanins, carotenoids, and others. The colour depends on the pH and the methylation or acylation of their hydroxyl group rings. Anthocyanins are located in the outer layers of the cells of different fruits such as blueberries, red grapes, raspberries, blackberries, strawberries, and many more. The main anthocyanins investigated by the scientific community are delphinidin, pelargonidin, cyanidin, peonidin, and malvidin [97].

Anthocyanins are widely used in the food industry as colour additives [98]; their positive effects on human health include tumour-growth inhibitors, circulatory system support, anti-inflammatory and antioxidant properties, and immune system support [99].

In general, flavonoids are currently being intensively investigated from a medical point of view for their beneficial properties for human health, such as enzyme inhibition, antimicrobial, anti-allergic, antioxidant, vascular, anti-tumour activity, etc. [100]. Flavonoids through direct inhibition of free radicals can prevent cell damage by forming more stable flavonoid radicals and less reactive free radicals [101].

Chen et al. [102] reported on the effects of lotus plumule flavonoids in alleviating inflammatory symptoms by inhibiting the biosynthesis and production of NO, PGE2 and TNF-α (inflammatory mediators) and proinflammatory cytokines such as IL-1β and IL-6. 

AL-Ishaq et al. [103] reported the beneficial effects of flavonoids in the fight against diabetes by influencing carbohydrate digestion, insulin secretion and signalling, fat deposition, and glucose uptake.

#### 3.5.7. Tannins

Several reports have shown that natural tannins and compounds distributed by various types of plants have beneficial effects on health by presenting antioxidant, hypoglycaemic, anti-tumoural, antibacterial, and hypoglycaemic properties [104]. Tannins are classified into the following three groups depending on their structure: hydrolysable tannins, condensed tannins, and compound tannins (Table 5) [105].

#### 3.5.8. Condensed Tannins

These types of tannins are considered oligomers or polymers which, depending on the hydroxylation pattern of the A and B rings of their flavan-3-ol units [106,107], are classified into prodelphinidins, procyanidins, and propelargonidins. These types of tannins possess high antioxidant potency by acting through hydrogen atom or single-electron transfer mechanisms, and are also noted for their anti-inflammatory, antimicrobial and anticarcinogenic properties [108,109,110].

An excess of these tannins causes changes in taste and an astringent feel to the food; hence, different methods have been developed to remove excess tannins, which can be done by physical, chemical, and biological means [111].

According to Laddha and Kulkarni [112], one of the most important types of condensed tannins is proanthocyanidin, which can be found in various foods. The same authors report that dark chocolate has a strong composition of catechin and epicatechin, and therefore, has potent antioxidant activity.

#### 3.5.9. Hydrolysable Tannins

Hydrolysable tannins contain various monosaccharides (oak, hazelnut, and quebracho) which have a high content of arabinose, glucose, fructose and glucose, but only those from vine bunches and nut galls contain fructose and glucose [113]. Through hydrogen bonds, hydrolysable tannins are able to interact with different cereals, but excess tannins in these products slow down or reduce the digestibility of protein and starch [114,115].

Gallotannin extracts, whose trade name is tannic acid (CAS number: 1401-55-4), depending on the plant source used for extraction are made with mixtures of polygalloylglucose esters or polygalloylquinic acid with a range in the number of galloyl molecules from 2 to 12 [116]. The galloyl units are linked by various polyols, catechins or tri-terpenoid units [117].

#### 3.5.10. Complex Tannins

This subclass of tannins has flavone as its basic unit and is found mainly in legumes, nuts, maize, rice, and tea. It is formed from the combination of an ellagitannin or gall tannin unit and a catechin. Its main positive effects on health include neuroprotective effects [118].

According to Molino et al. [119], several tannins extracted from wood showed positive biological effects in humans and animals, including anti-tumour, antidiabetic, antibacterial, antifungal, and anti-mutagenic properties.

Another article [120] reported the ability of tannins to form tannates, which are stable compounds formed from the binding of tannins to metal ions present in the body; this can be beneficial or harmful to human health, as they can be used to deal with overexposure to heavy metals, but their daily overconsumption can lead to nutrient deficiencies such as calcium and iron, causing osteoporosis and anemia.

### 3.6. Methylxanthines (Theophylline, Caffeine, and Theobromine)

Methylxanthines are compounds of organic heterocyclic origin that are derived from purine; they are structured by coupled pyrimidinedione and imidazole rings (Table 6) [121] and originate naturally in different products such as coffee, chocolate, tea, soft drinks, mate, and energy drinks, among others [122,123,124]. The main components of methylxanthines are caffeine (CAS number: 58-08-2), theophylline (CAS number: 58-55-9), and theobromine (CAS number: 83-67-0). Among the main biological functions of methylxanthines are their anti-asthmatic, analgesic, energetic, chronoprotective, anti-inflammatory, antioxidant, and neuroprotective properties [125,126,127,128].

#### 3.6.1. Theophylline

Theophylline (1,3-dimethylxanthine) is derived from a methylated xanthine [129,130], the extraction of which is mainly from *Camellia sinensis* L. and *Ilex Paraguariensis*. Its main biological properties are the decrease of metastasis and inflammation and resistance to therapy in cancer cells. According to Pérez-Pérez et al. [131] theophylline inhibits the PI3K pathway which is a cancer activator that promotes metastasis and resistance to treatment; it is also able to inhibit the expression of inflammatory genes by activating the histone deacetylase 2 protein. 

#### 3.6.2. Theobromine

Theobromine and theophylline are present in the tea plant, while the former is a precursor of caffeine biosynthesis, the latter is a caffeine biodegrader [132]. Theobromine originates from xanthine methylation, with a strong adenosine receptor antagonist and non-selective phosphodiesterase inhibitory activity, it increases adenosine monophosphate in the nervous system [133,134]; in turn, theobromine exhibits bronchodilator, diuretic, and antitussive effects and influences angiogenesis in tumour growth [135]. According to Ejuh et al. [136], this compound inhibits the crystallization of uric acid with a great capacity for the treatment and clinical prevention of uric acid-influenced nephrolithiasis.

#### 3.6.3. Caffeine

Currently, the most studied and consumed methylxanthine is caffeine, which can be found in various plants such as tea, coffee, cola, and guarana, and various products such as soft drinks, energy drinks, and chocolate, among others [137,138]. Caffeine is capable of forming the natural metabolites theophylline and theobromine [139]. Among its beneficial effects for humans are stimulation of the nervous system, analgesic effects, diuresis, psychomotor enhancement [140], and gastric acid secretion, as well as negative effects such as nausea, anxiety, increased blood pressure, tremors, and nervousness [141,142]. Caffeine increases the capacity for the occurrence of chromosomal mutations and potentiates cytotoxic, mutagenic, and carcinogenic activities in different animal cells [143].

Due to the wide range of studies carried out that are focused on the biological effects of caffeine on different molecular targets, the following stand out: its antagonist activity on adenosine receptors, the inhibitory effects on phosphodiesterases, the sensitization of cannulae sensitive to ryanodine for the release of calcium in the sarcoplasmic and endoplasmic reticulum, as well as its antagonist activity on GABAA receptors [144]. 

According to an experimental study, caffeine consumption increased blood pressure and heartbeat [145]. These effects of caffeine on blood pressure were more visible from the consumption of 205 mg per day, where the greatest effects can be seen in the elderly, the hypertensive population, and those who have never consumed caffeine [146].

Some examples of the plants and their bioactive compounds are reported in Table 7, with a focus on which biocompounds had the ability to benefit the skin cell culture.

## 4. Antioxidants as Reactive Oxygen Species Antagonists in Skin Conditions—Possible Mechanism

There are a lot of harmful oxygen byproducts in the aerobic environment. The organism has developed antioxidant defence mechanisms to protect it from adverse effects. Antioxidants are “any substance that directly scavenges reactive oxygen species (ROS) or indirectly acts to upregulate antioxidant defences or inhibit ROS production” [39]. Still, antioxidants may also undergo further oxidation and intramolecular hydrogen bonding to generate a new, more stable radical [160]. Furthermore, antioxidants can control gene expression, which causes the nuclear factor erythroid 2-related factor 2 (Nrf-2) to move from the cytosol to the nucleus after splitting apart from its inhibitor, the Kelch-like erythroid cell-derived protein 1. After entering the nucleus, Nrf-2 may bind antioxidant response elements and trigger the transcription of genes related to stress response, including NAD(P)H: quinone acceptor oxidoreductase 1, glutathione S-transferase, and heme-oxygenase-1 [161,162,163].

**Figure 1:**
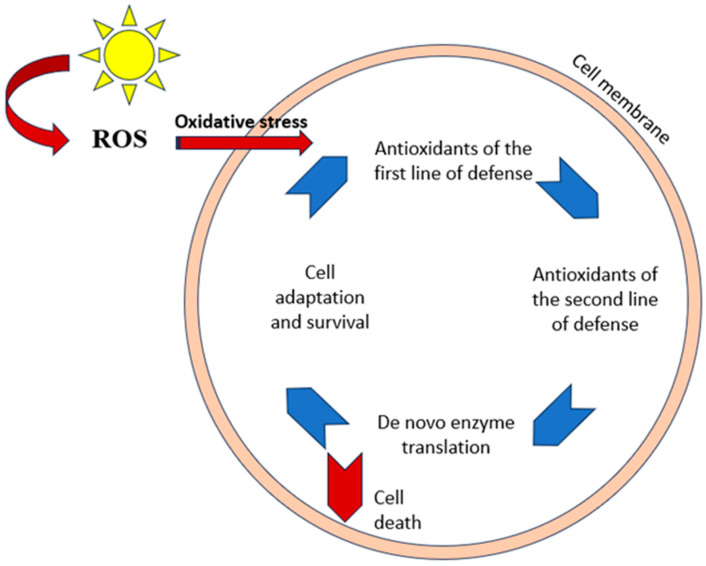
Antioxidant response of the cell after damage from oxidative stress. ROS levels rise, and oxidative stress is brought on by UV exposure.

The cell’s defence mechanism against oxidative stress is made up of an interconnected network of several antioxidants (e.g., superoxide dismutase, catalase, glutathione peroxidase, transferrin, and caeruloplasmin) that function in various ways and at various degrees (first, second, and third lines of defence) [163] (Figure 1).

ROS production is inhibited by endogenous antioxidants, and propagation reactions are suppressed by the combined action of exogenous and endogenous antioxidants. Enzymes generated from scratch restore damage caused in the cells. Ultimately, the cell will survive due to an adaptation process if the cooperation of various networks associated with antioxidants is able to resist oxidative stress damage. If the stress is continuous, the cell will eventually die [164].

## 5. Conclusions

For many years, skincare treatments derived from plants have been used. Natural components are still widely used in various novel formulations for skin care, cleansing, and protection (natural products often enhance their action when combined with each other and not simply isolated and concentrated). For medicinal and cosmetic uses, individual active chemicals and compounds found in plants are used; they are often taken in the form of extracts made from different plant tissues. The reason why plant extracts are employed is their ability to shield the skin from damaging external or internal causes. The primary advantages of using natural substances are their antioxidant qualities and their capacity to shield against oxidative stress-related skin problems. Plant extracts’ ability to defend against UV radiation is particularly significant since UV-induced photo-oxidative damage to cellular lipids, proteins, and DNA is linked to early skin ageing and the emergence of skin cancer. Plants have much to offer regarding skin care, but further studies and clinical proof are required since many of these extracts’ efficacy is still up for debate. Moreover, there are still a lot of active molecules to be found, and natural compounds made from plant extracts make for a fascinating area of study. A great challenge of new aromatic and therapeutic plants that enhance the quality of plant-based goods may be discovered in the future.

## Figures and Tables

**Table 1:** Molecular structures, IUPAC names and CAS numbers for Vitamin A, Vitamin C, Vitamin E, and Coenzyme Q10.

Bioactive Compound	Vitamin A	Vitamin C	Vitamin E	Coenzyme Q10
Molecular structure	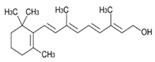	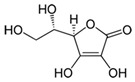	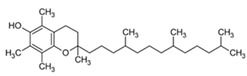	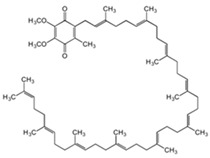
IUPAC name	3,7-dimethyl-9-(2,6,6-trimethylcyclohex-1-yl) nona-2,4,6,8-tetraen-1-ol	(5R)-[(1S)-1,2-dihydroxyethyl]-3,4-dihydroxyfuran-2(5H)-one	(2R)-2,5,7,8-tetramethyl-2-[(4R,8R)-4,8,12-trimethyltridecyl]-3,4-dihydrochromen-6-ol	2-[(2E,6E,10E,14E,18E,22E,26E,30E,34E)-3,7,11,15,19,23,27,31,35,39-decamethyltetraconta-2,6,10,14,18,22,26,30,34,38-decaenyl]-5,6-dimethoxy-3-methylcyclohexa-2,5-diene-1,4-dione
CAS number	68-26-8	50-81-7	59-02-9	303-98-0

**Table 2:** Molecular structures, IUPAC names and CAS numbers for resveratrol, piceatannol, pinosylvin, and pterostilbene.

Bioactive Compound	Resveratrol	Piceatanol	Pinosylvin	Pterostilbene
Molecular structure	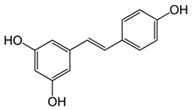	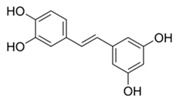	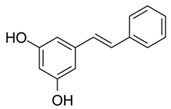	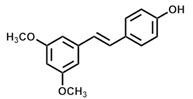
IUPAC name	5-[(E)-2-(4-hydroxyphenyl)ethenyl]benzene-1,3-diol	4-[(E)-2-(3,5-dihydroxyphenyl)ethen-1-yl]benzene-1,2-diol	5-[(1E)-2-phenylethen-1-yl]benzene-1,3-diol	4-[(E)-2-(3,5-dimethoxyphenyl)ethen-1-yl]phenol
CAS number	501-36-0	10083-24-6	22139-77-1	537-42-8

**Table 3:** Molecular structures, IUPAC names and CAS numbers for p-hydroxybenzoic, protocatechuic, vanillic, gallic, syringic, caffeic, p-coumaric, ferulic and sinapic acids.

Bioactive Compound	Molecular Structure	IUPAC Name	CAS Number
P-hydroxybenzoic acid	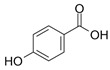	4-hydroxybenzoic acid	99-96-7
Protocatechuic acid	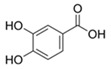	3,4-dihydroxybenzoic acid	99-50-3
Vanillic acid	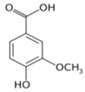	4-hydroxy-3-methoxybenzoic acid	121-34-6
Gallic acid	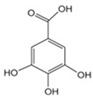	3,4,5-trihydroxybenzoic acid	149-91-7
Syringic acid	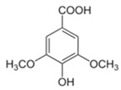	4-hydroxy-3,5-dimethoxybenzoic acid	530-57-4
Caffeic acid	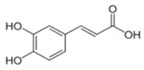	(2E)-3-(3,4-Dihydroxyphenyl)prop-2-enoic acid	331-39-5
P-coumaric acid	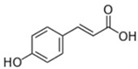	(2E)-3-(4-hydroxyphenyl)prop-2-enoic acid	501-98-4
Ferulic acid	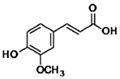	(2E)-3-(4-hydroxy-3-methoxyphenyl)prop-2-enoic acid	537-98-4
Sinapic acid	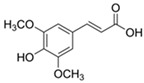	3-(4-hydroxy-3,5-dimethoxyphenyl)prop-2-enoic acid	530-59-6

**Table 4:** Molecular structures for Flavones, Flavonols, Flavanols, Flavanones, Isoflavones, and Anthocyanins.

Bioactive Compound	Molecular Structure
Flavones	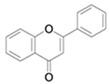
Flavonols	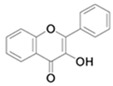
Flavanols	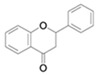
Flavanones	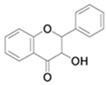
Isoflavones	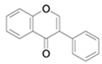
Anthocyanins	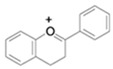

**Table 5:** Molecular structures for tannins, condensed tannins, and hydrolysable tannins.

Bioactive Compound	Molecular Structure
Tannins	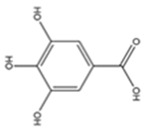
Condensed tannins	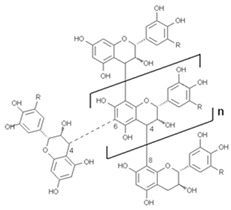
Hydrolysable tannins	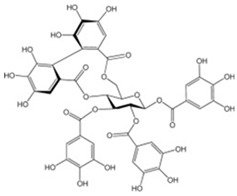

**Table 6:** Molecular structures, IUPAC names and CAS numbers for theophylline, theobromine, and caffeine.

Bioactive Compound	Molecular Structure	IUPAC Name	CAS Number
Theophylline	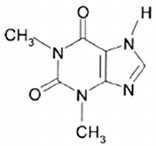	1,3-dimethyl-7H-purine-2,6-dione	58-55-9
Theobromine	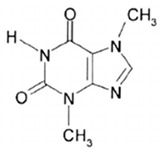	3,7-dimethyl-3,7-dihydro-1H-purine-2,6-dione	83-67-0
Caffeine	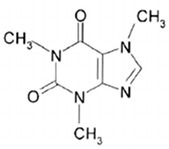	1,3,7-trimethyl-3,7-dihydro-1H-purine-2,6-dione	58-08-2

**Table 7:** Examples of in vitro studies of plant extracts and their biocompound effects on skin.

No	Botanical	Biocompound	Effects on Skin	Cells Type	Ref.
1	*Butyrospermum parkii*	Flavan-3-ols (catechin)	antioxidant, UV-induced skin damage prevention, collagen synthesis activation, matrix metalloproteinases inhibition	HaCaT	[1]
2	*Glycyrrhiza glabra* (licorice) leaf extract	Isoflavones (wighteone)	antioxidant, UV-induced skin damage prevention, anti-inflammatory, and estrogenic effects	HaCaT	[1]
3	*Simmondsia chinensis*	Tannins	antioxidant, astringent, wound-healing promotion	HaCaT	[1]
4	*Helianthus annuus*	Hydroxycinnamic acid derivatives (chlorogenic, acid, caffeic acid, ferulic acid)	antioxidant, UV-induced skin damage prevention, MMP inhibition, anti-inflammatory, anti-tyrosinase	HaCaT	[1]
5	*Theobroma cacao*	Flavan-3-ols	antioxidant, UV-induced skin damage prevention, collagen synthesis activation, MMP inhibition	HaCaT	[1]
6	*Calendula officinalis*	Flavonols (quercetin, rutin, narcissin, isorhamnetin, kaempferol)	antioxidant, cell longevity increase	HaCaT	[1]
7	*Glycyrrhiza glabra*	Dihydroxyflavanones (glabranin, licoflavanone)	antioxidant, anticancer	HaCaT	[1]
8	*Citrus limon*	Flavonoids	anti-inflammatory, antimicrobial, anticancer	HaCaT	[147]
9	*Verbena officinalis*	Flavonoids	antiproliferative and anticancer	HCT-116	[148]
10	*Symphytum officiale*	n.d	boost the regenerative power of epidermal stem cells and their ability to build new tissue;	Callus culture	[149]
11	*Camellia sinensis*	Flavonoids glycosides	antioxidant, anti-ageing, photoprotective properties	keratinocyte	[150]
12	*Papaver roheas*	Flavonoids (anthocyanins), quercetin	antioxidant and anti-inflammatory	fibroblasts and keratinocytes	[151]
13	*Punica granatum*	Flavonoids (anthocyanins), quercetin	antioxidant and anti-inflammatory	fibroblasts and keratinocytes	[151]
14	*Clitoria ternatea*	Flavonoids (anthocyanins), quercetin	antioxidant and anti-inflammatory	fibroblasts and keratinocytes	[151]
15	*Carthamus tinctorius*	Carthamin, Quercetin	antioxidant and anti-inflammatory	fibroblasts and keratinocytes	[151]
16	*Gomphrena globosa*	Betacyanins, quercetin	antioxidant and anti-inflammatory	fibroblasts and keratinocytes	[151]
17	*Rubus idaeus*	n.d	anti-ageing, antioxidant	Keratinocyte	[152]
18	*Rosmarinus officinalis*	Flavonoids, polyphenols	antioxidant, anticancer, anti-ageing, anti-inflammatory	Keratinocyte	[153]
19	*Cannabis*	Flavanols and flavones	anti-ageing	Keratinocytes	[154]
20	*Epilobium angustifolium*	Flavonoids	anti-ageing and anti-inflammatory properties	HaCaT	[155]
21	*Schisandra chinensis*	Flavonoids (quercetin, rutinoside)	radiation-protective, anti-ageing, antioxidant, anti-allergic and anti-inflammatory	HaCaT	[156]
22	*Curcuma longa*	Flavonoids (rutin and quercetin-O-hexose)	anti-inflammatory and antioxidant	HaCaT	[157]
23	*Dendrobium officinale*	Stilbenoid	antioxidant, anticancer	HEK-293	[158]
24	*Cocos nucifera*	caprylic acid, capric acid, lauric acid, stearic acid, linoleic acid	anti-inflammatory and skin protective	Keratinocytes	[159]

n.d—not determined.
